# Altered network connectivity and global efficiency in tourette syndrome: insights into sensorimotor integration

**DOI:** 10.1016/j.nicl.2025.103845

**Published:** 2025-07-13

**Authors:** Julia Schmidgen, Theresa Valentine Heinen, Felix Schmitt, Kerstin Konrad, Stephan Bender

**Affiliations:** a University of Cologne, Univ. Hosp Cologne, Department of Child and Adolescent Psychiatry, Germany; b Section Child Neuropsychology, Department of Child and Adolescent Psychiatry, Psychosomatics and Psychotherapy, Universitätsklinik RWTH Aachen, Germany; c JARA-BRAIN Institute II, Molecular Neuroscience and Neuroimaging, Forschungszentrum Jülich GmbH and RWTH Aachen University, Germany; d Computational Systems Neuroscience, Institute of Zoology, University of Cologne, Germany

**Keywords:** Tourette syndrome, Sensorimotor integration, Theta connectivity, Phase locking values, Global efficiency

## Abstract

This study investigates the role of theta connectivity in network mechanisms related to perception–action-binding, sensorimotor integration, and motor preparation in children with Tourette’s Syndrome (TS). High-density EEG data were collected from 21 children with drug-naïve TS and 21 age-matched healthy controls during a task combining an informative warning stimulus (S1) with a behaviorally relevant imperative stimulus (S2). Event-related phase synchronization and global efficiency were calculated to analyze stimulus processing and identify neural networks responsible for integrating sensory information with motor preparation processes. Results revealed widespread alterations in theta-band connectivity in TS, with patients exhibiting reduced connectivity and impaired network efficiency during S1 processing. S2 processing revealed subtler group differences than S1, manifesting as shifts in network organization rather than overall loss in connectivity strength. Remarkably, global efficiency during S2 processing remained intact in the TS group. In both groups, higher global efficiency during S2 correlated with faster reaction times, highlighting a direct link between network efficiency and motor response speed. This suggests that, despite altered sensory processing during information integration of the warning stimulus, motor execution mechanisms remain preserved in TS. The reduced connectivity during S1 processing may represent a compensatory mechanism aimed at weakening perception–action binding, potentially preventing premature motor output and aiding in tic control. These findings support the view of TS as a network disorder extending beyond traditional cortico-striato-thalamo-cortical circuits and suggest potential targets for interventions to modulate network efficiency and compensatory mechanisms in TS.

## Highlights

• Children with Tourette Syndrome show reduced theta connectivity during sensory integration.
• Motor execution network efficiency is preserved in Tourette Syndrome.
• Higher global efficiency correlates with faster reaction time.
• Findings support Tourette Syndrome as a network disorder with possible compensatory mechanisms.

## 1 Introduction

Motor control relies on a complex processing network including various brain structures and neural pathways. Disruption of these highly organized, dynamic networks can lead to movement disorders, such as Tic disorders or Tourette’s syndrome (TS). TS is a complex neurodevelopmental childhood-onset disorder characterized by chronic motor and vocal tics. Tics are nonrhythmic, repetitive, involuntary movements or vocalizations occurring in bouts for a limited duration (Leckman et al., 2014). Tics can be simple or complex, ranging from sudden movements or sounds involving a limited number of muscle groups to coordinated action sequences. TS is diagnosed when multiple motor tics and at least one vocal tic have been present for a minimum of one year. The severity and frequency of tics fluctuate over time, often peaking in early adolescence and declining steadily for most individuals as they progress through adolescence (Ricketts et al., 2022). TS often comes along with various comorbidities such as attention deficit hyperactivity disorder (ADHD) and obsessive–compulsive disorder, significantly impacting quality of life (Cravedi et al., 2017, Eapen et al., 2016, Kumar et al., 2016).

A key challenge in managing TS is the limited understanding of its pathophysiology, especially considering the complexity of the underlying mechanisms and the involvement of multiple brain regions (Albin, 2018, Yael et al., 2015). The pathophysiology of TS is thought to result from a complex interplay among different neural systems. Motor control deficits underlying tic generation have been closely linked to disruptions in the cortico-striato-thalamo-cortical circuits. Recent research has focused on the role of sensorimotor integration in TS, with emerging evidence indicating abnormalities in how sensory information is processed and integrated with motor output (Houghton et al., 2014). Individuals with TS frequently report premonitory urges and somatic hypersensitivity, suggesting potential impairments in sensorimotor integration. Furthermore, the concept of perception–action binding has gained increasing attention in TS research, with evidence suggesting that patients with TS exhibit an enhanced coupling between sensory stimuli and motor responses (Friedrich et al., 2021, Kleimaker et al., 2020).

Theta-band activity is strongly related to processes involving sensorimotor integration and perception–action binding (Beste et al., 2023, Cruikshank et al., 2012, Tomassini et al., 2017, Wendiggensen et al., 2023). Especially long-range theta connectivity is suggested to be a crucial factor for effective cognitive processing and coordination of task-dependent activity (Lisman and Jensen, 2013, Mizuhara et al., 2004, Von Stein and Sarnthein, 2000). Therefore, the investigation of theta-band connectivity related to stimulus-evoked network changes is of particular interest in the present study, as alterations in these networks may contribute to the abnormal sensorimotor integration and perception–action binding observed in TS.

Efficient coordination of brain activity requires intricate interactions between anatomically separated neural populations (Horwitz, 2003). Investigations of these interactions reveal complex networks underlying brain functions. Synchronization of anatomically separated neuronal populations is considered to play an important role in coordinating information and indicating enhanced functional connectivity (Buehlmann & Deco, 2010). Phase locking values (PLVs) provide a metric to quantify the degree of synchronization between neural oscillations across different brain areas and to investigate dynamic network communication (Lachaux et al., 1999). PLVs measure the consistency of phase differences between oscillatory signals recorded from different brain regions. High PLVs (close to 1) suggest robust communication between neuronal populations, while low PLVs (close to 0) indicate weak or no functional interaction. To investigate task-related changes in network synchronization, relative PLVs (rPLVs) can be calculated to assess changes in phase locking relative to baseline synchronization. Only few studies examined theta connectivity in TS with preliminary findings pointing to altered network dynamics that vary by context. Takacs et al. (2024), using resting-state EEG, found that individuals with TS show increased adaptability in theta network architecture, potentially reflecting hyper-learning of sensorimotor patterns. In contrast, Loo et al. (2019) used a task-based EEG paradigm and observed reduced theta connectivity during voluntary movements, suggesting impaired task-specific coordination. Wang et al. (2025) described disrupted theta-related connectivity between the supplementary motor area (SMA) and basal ganglia circuits, supporting its role as a therapeutic target via rTMS.

The present study aims to investigate the role of theta connectivity in network mechanisms related to perception–action-binding, sensorimotor integration, and motor preparation. We calculated phase synchronization from high-density EEG data of children with TS and age-matched healthy controls. To achieve a more detailed analysis of stimulus processing, we employed a Contingent Negative Variation task paradigm that combined an informative warning stimulus (S1, indicated by an arrow pointing to either the left or right) with a behaviorally relevant imperative stimulus (S2). The Contingent Negative Variation is a slowly rising negative potential measured by EEG that reflects anticipation of the upcoming imperative Stimulus. Since S2 follows S1 with a constant delay, the informative cue allowed participants to prepare a specific motor response in advance. This paradigm enables the investigation of the neural networks responsible for integrating sensory information with motor preparation processes.

We hypothesize that (i) theta-band connectivity networks will be altered in children with TS; (ii) these alterations will be most pronounced during S1 stimulus processing, as this phase primarily involves sensorimotor integration and perception–action binding; and (iii) the strength of theta connectivity during the cue processing phase will predict subsequent task performance in both groups, reflecting the role of theta-band activity in cognitive processing and task-dependent coordination. Investigating the interactions among neural networks involved in sensory integration not only deepens our understanding of tic pathophysiology but also may guide the development of targeted therapeutic interventions and aid in identifying neurophysiological biomarkers for TS.

## 2 Material and methods

### 2.1 Subjects

The study included 21 drug-naive TS patients and 21 healthy controls (CO) subjects aged 7 to 13 years. Tic patients were individually matched with healthy controls based on age and gender. The maximum age difference between any matched pair of tic patients and control subjects was 0.4 years. There were no gender differences between any matched pairs. This matching procedure ensured that both age- and gender-related differences were minimized between the two groups. One subject with TS had to be excluded from further analysis due to poor data quality (for details, see 2.6). To preserve the integrity of the matched-pair design, the corresponding CO subject was also removed from the analysis. Detailed sample characteristics are provided in Table 1. All subjects were right-handed, assessed by the Edinburgh Handedness Scale (Oldfield, 1971). To ensure diagnostic accuracy, we conducted the K-DIPS structured clinical interview with all parents and with adolescents aged 12 and older. This process confirmed the diagnoses in patients and verified the absence of neuropsychiatric conditions in healthy controls. Participants with (i) a full-scale IQ below 70 as measured by WISC-V, (ii) individuals with a history of epilepsy or other central nervous system (CNS) disorders, (iii) those born prematurely before 32 weeks gestation, (iv) individuals with uncorrectable visual impairments, (v) and those with current or previous use of psychoactive drugs were excluded. Furthermore, we did not include any tic patients with comorbid ADHD in our sample. This allows for analysis of theta-band connectivity networks in drug-naive patients and controls and their abnormalities specifically related to tics. All participants and their legal guardians provided informed written consent/assent in accordance with the Declaration of Helsinki. The local ethics committee reviewed and approved the study protocol.

**Table 1:** Sample demographics.

Sample characteristics	TS (N = 20)	Control (N = 2ß)	Statistic	*p*
Age [M ± SD; range]	10.6 (±1.8; 7.4–13.2) years	10.8 (±1.7; 7.8–13.2)	t(38) = −0.35	0.73
Male gender [n (%)]	16 (80)	16 (80)	X^2^ (1) = 0.00	1.0
Yale Global Tic Severity Score [M ± SD]	33.65 (±13.21)			
Age at tic onset (M ± SD)	5.03 (±1.66) years			
Duration of tics [years (M ± SD; range)]	5.41 (±2.52)			
Comorbid ADHD [n (%)]	0			
Comorbid OCD [n (%)]	0			

*Note.* M = Mean; SD = Standard deviation, ADHD = Attention deficit hyperactivity disorder, OCD = Obsessive compulsive disorder.

### 2.2 Experimental procedure

The software package *Presentation* (Version 10.3, Neurobehavioral Systems Inc., Albany, CA) was used to generate a task-related program with alternating visual stimuli displayed on a monitor at a distance of 90 cm from the subjects. To prevent any distractions, the light was dimmed, and the noise level was reduced as much as possible. To minimize distracting eye movements, a fixation cross was displayed between visual stimuli. Subjects sat in a comfortable position on a skid-proof chair, reducing muscle activity as much as possible to prevent interfering contractions.

### 2.3 Behavioral task paradigm

**Fig. 1:**
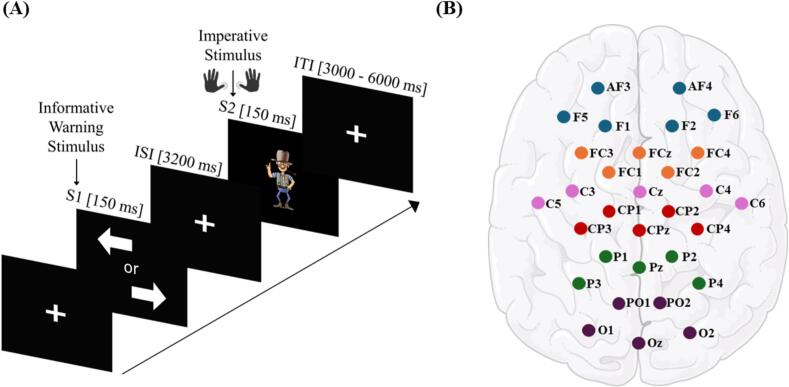
Experimental design (A) Contingent negative variation task paradigm with a directional warning stimulus S1 and a behaviourally relevant imperative stimulus S2. (B) Electrode locations with regions of interest and specific electrodes used for data analysis marked in blue (DLPFC), orange (SMA/PMA), pink (M1), red (S1), green (PPC), and purple (POC). Sensor locations were plotted by using MNE-Python (Gramfort et al., 2013). (For interpretation of the references to colour in this figure legend, the reader is referred to the web version of this article.)

Participants completed a visual Contingent Negative Variation task paradigm (Fig. 1) consisting of 50 trials per response side. The warning stimulus (S1) appeared as a black arrow on a white background, pointing either left or right. The imperative stimulus (S2) was depicted as a colored sheriff on a black background. Both stimuli were displayed for 150 ms, with a fixed interstimulus interval of 3.05 s. Intertrial intervals varied pseudorandomly between 3 and 6 s. The warning stimulus indicated the required response side, with arrows presented in a pseudorandomized order. Participants were instructed to respond as quickly as possible to the imperative stimulus (S2) by pressing the corresponding button − “Ctrl” for left and “Enter” on the numeric keypad for right − using their left or right thumb on a German standard keyboard.

### 2.4 Electroencephalography

EEG was recorded using a 64-channel BrainAmp system (BrainProducts, Munich, Germany) and Brain Vision Recorder software (BrainProducts). Elastic EEG caps with direct current sintered Ag/AgCl disc electrodes (BrainProducts) were selected based on head sizes. Electrodes were named based on their location on the scalp, consistent with the international 10–20 system, electrode impedances were kept below 5 kΩ. Additionally, EOG electrodes were positioned under the left and right eyes and on the nasion. The sampling rate was set to 5000 Hz, electrode Cz was used as the online recording reference.

### 2.5 Electromyography

Surface EMG (compound muscle action potential) was recorded using self-adhesive silver-silver chloride electrodes in a belly tendon montage, respectively for the left and right hand. To record thumb movement, active electrodes were placed on the adductor pollicis muscle and reference electrodes were attached to the exterior proximal phalanx of the thumb. The ground electrode was placed on the inner forearm. The EMG was recorded using the bipolar BrainAmp ExG amplifier (Brain Products, Munich, Germany), which was synchronized with the EEG recordings.

### 2.6 Signal preprocessing

The raw EEG data were preprocessed using MNE-Python (v0.22) within Python (v3.8.8). To reduce file size and computational load, data were downsampled to 200 Hz and digitally filtered using an FIR 50 Hz notch filter. The Cz online reference channel was reconstructed via spherical spline interpolation. EEG signals were then re-referenced to a common average reference and epoched around the imperative stimulus (S2) for both left and right button press conditions using MNE metadata. Epochs began 0.5 s before the warning stimulus (S1) for baseline computation and extended until 3 s after S2, capturing post-processing activity (total duration: 6.7 s, 1341 data points). Only trials in which participants responded correctly within 1.5 s following S2 were included in further analyses. To minimize the influence of slow drift effects on EEG data, a linear DC detrend was applied. Visual inspection before and after detrending confirmed that the overall signal shape and characteristics remained unaffected. For reproducibility, artifact rejection and trial exclusion were performed automatically using ‘Autoreject’, implemented in Python (Jas et al., 2017). This method employs cross-validation with a robust error metric to estimate rejection thresholds, detecting and correcting outlier data segments for each sensor. Independent component analysis was then conducted, and components associated with eye blinks, muscle activity (e.g., jaw clenching, swallowing), or other artifacts were automatically identified and removed using *MNE-ICALabel*. Finally, epoched and cleaned data were baseline-corrected using a reference window from 500 to 0 ms before the warning stimulus (S1).

After excluding trials with errors or strong artifacts (e.g., excessive movement), only participants with at least 35 valid trials per hand were included in further analysis. Based on these criteria, data from one participant with TS was removed. To preserve the matched-pair design, the corresponding CO participant was also excluded. This ensured that the study maintained its age- and gender-matched integrity throughout the analysis. Importantly, the number of valid trials did not differ between groups (TS: M = 83.05, SD = 9.76; CO: M = 82.45, SD = 11.18; t(19) = 0.86, p = 0.399, d = 0.19).

To minimize the effects of volume conduction, epoched data were transformed using current source density. Given the low spatial resolution of EEG, electrical signals from a single neural source can spread across multiple scalp electrodes (Nunez & Srinivasan, 2006). This diffusion can create the illusion of widespread synchronous or phase-locked activity, even when the signal originates from a single source. By applying Surface Laplacian spatial filtering, current source density enhances the localization of brain activity, effectively reducing the influence of distant sources (Tenke & Kayser, 2012).

### 2.7 Phase-locking connectivity analysis

Epoched, cleaned, and reference-free data were transformed into the time–frequency domain using the Morlet wavelet transform. The analysis focused on the theta frequency range (4–7 Hz; 1 Hz increments) to capture oscillatory activity linked to attentional control, sensorimotor integration, and perception–action binding in children (Beste et al., 2023, Cruikshank et al., 2012, Tomassini et al., 2017, Wendiggensen et al., 2023). To test whether significant synchronized activity was specific to the theta band, we conducted parallel analyses in the alpha frequency range (8–13 Hz). For subsequent phase-locking analysis, we used a modified version of the Dynamic Synchronization Toolbox (DST;(Rosjat & Daun, 2022)) implemented in MATLAB (v.R2022b, MathWorks, Natick, MA, USA).To ensure compatibility with the DST data structure, phase data arrays were reformatted. The final dataset was structured with dimensions corresponding to channels, time, trials, and frequencies, and saved as a MATLAB matrix. The instantaneous Phase is defined as

$$\mathrm{PLVij}\left(t\right)=\frac{1}{N}\left|\sum_{n=1}^{N}e^{-i(\varphi_{i}\left(t,n\right)-\varphi_{j}\left(t,n\right))}\right|$$

where $\varphi_{i}\left(t,n\right)$ and $\varphi_{j}\left(t,n\right)$ represent the instantaneous phases of signals i and j at time *t* in trial *n* with *N* for the total number of trials. Since our study focused on event-related phase-locking changes, PLVs were normalized relative to mean PLV baseline values [−3700, −3200] and denoted as “relative phase-locking values” (rPLV):

$$r{\mathrm{PLV}}_{\mathrm{ij}}\left(t\right)=\frac{{\mathrm{PLV}}_{\mathrm{ij}}\left(t\right)-\overset{¯}{{\mathrm{PLV}}_{\mathrm{ij}}}}{\overset{¯}{{\mathrm{PLV}}_{\mathrm{ij}}}}$$

Based on previous literature examining cue processing and perception–action binding, we focused our analysis on two critical time windows: [−3200, −2700] ms relative to the imperative stimulus for cue processing, and 500 ms following the imperative stimulus for initial motor preparation and response execution. These time windows were chosen to specifically capture stimulus-evoked sensorimotor, cognitive processing, and perception–action binding rather than classical Contingent Negative Variation dynamics, which typically focus on activity between S1 and S2. While EEG studies often analyze early (550–750 ms post-S1) or late phases of the Contingent Negative Variation (response preparation just before S2), our approach prioritizes the neural mechanisms underlying stimulus processing and the initiation of motor responses. This distinction is crucial, as the study aims to investigate tic-related alterations in phase synchronization during cue and imperative stimulus processing, rather than sustained activity (Morand-Beaulieu et al., 2015, Rothenberger and Kemmerling, 1982, van Woerkom et al., 1994).

### 2.8 Network of interest

To investigate potential deficits in TS related to sensorimotor integration, visual processing, motor preparation, and control, we identified key brain regions for analysis. Given that different neural regions contribute to various aspects of these processes, multiple regions of interest were defined (Makeig et al., 2002). This approach enables a comprehensive examination of hierarchical processing, functional specialization, and network interactions during stimulus processing and sensorimotor integration. Table 2 provides an overview of the selected brain areas, their associated functions, and corresponding electrode positions (see also Fig. 1B for graphical illustration).The dorsolateral prefrontal cortex (DLPFC) is implicated in cognitive control, top-down attention allocation and flexible adjustment of behavior to changing task demands (Brosnan and Wiegand, 2017, Cieslik et al., 2013, Fitzgerald et al., 2009). The supplementary motor area and premotor areas (SMA/PMA) are crucial for action inhibition and higher-order motor planning (Goldberg, 1985, Nachev et al., 2007). The primary motor cortex (M1) is the principal cortical area for the execution of voluntary movements, especially fine motor control of the hand (Gerloff et al., 1998, Lemon, 2008), while the primary somatosensory cortex (S1) is essential for processing motor-related sensory input and feedback (Borich et al., 2015, Schneider, 2020). The posterior parietal cortex (PPC) contributes to spatial attention and the transformation of sensory information into motor commands (Andersen and Cui, 2009, Freedman and Ibos, 2018). Finally, the parieto-occipital cortex (POC) is involved in the integration of visual information, facilitating perceptual processing and visuomotor coordination (Babiloni et al., 2005, Frank et al., 2020). These areas of interest were defined based on established functional neuroanatomy and their relevance to the processes engaged by the motor task paradigm.

**Table 2:** Regions of interest.

Brain areas	Involvement	Electrode position	References
Dorsolateral Prefrontal Cortex (DLPFC)	Cognitive control, attentional processing, motor preparation	F5, F1, AF3; AF4, F2, F6	(Fitzgerald et al., 2009, Kaneko et al., 2024)
SMA and Pre-motor Areas (SMA/PMA)	Action inhibition, motor control	FC4, FC2, FCZ, FC1, FC3	(Ahangama et al., 2023, Puzzo et al., 2010)
Primary Motor Cortex (M1)	Motor control of hand movement	C5, C1, Cz, C4, C6	(Jessy, 2009, Puzzo et al., 2010, Yuan et al., 2010)
Primary Somatosensory Cortex (S1)	sensorimotor integration and processing of sensory information	CP3, CP1, CPz, CP2, CP4	(Insausti-Delgado et al., 2021, Kim et al., 2021)
Posterior Parietal Cortex (PPC)	Sensorimotor integration, attention allocation	P3, P1, Pz, P2, P4	(Lin et al., 2015, SanMiguel et al., 2010)
Parieto-Occipital Cortex (POC)	Visual processing, integration of visual information	PO1, PO2, O1, Oz, O2	(Boutonnet et al., 2013, Pouryazdian and Erfanian, 2009)

### 2.9 Euclidean distance

We calculated the time-resolved Euclidean distance, denoted as *d(t)*, to quantify differences between the functional connectivity networks of the TS and CO groups. Functional connectivity networks were constructed by averaging the within- and between-area connections for each group. The Euclidean distance *d(t)* was then computed using the group-averaged adjacency matrices, reshaped into vectors $A_{\mathrm{TS}}\left(t\right)$ and $A_{\mathrm{CO}}\left(t\right)$. The formula for *d(t)* is as follows:

$$d\left(t\right)={\|A_{\mathrm{TS}}\left(t\right)-A_{\mathrm{CO}}\left(t\right)\|}_{2}$$

This measure quantifies the dynamic differences in functional connectivity between groups over time.

Furthermore, within-group variability was assessed by computing the variance of the Euclidean distance between each individual’s connectivity pattern and their respective group mean. This analysis provides insight into the consistency of functional connectivity within each group and highlights potential heterogeneity in network organization.

### 2.10 Principal component analysis

We performed PCA on the functional connectivity networks of the TS and CO groups. Connectivity networks were constructed by averaging within- and between-region connections. The group average adjacency matrices were reshaped into vectors $A_{\mathrm{TS}}\left(t\right)$ and $A_{\mathrm{CO}}\left(t\right)$ and concatenated. Prior to PCA, the data was mean-centered and scaled to unit variance. The resulting eigenvectors were extracted and reshaped to the adjacency matrix.

### 2.11 Classification – linear discriminant analysis

To assess distinction between TS and CO conditions based on functional connectivity patterns, we applied a machine learning classification approach. This analysis was not aimed at identifying TS biomarkers, as the data quality and sample size were insufficient. Instead, we explored whether group differences in connectivity were detectable and whether the data were separable within a given time window. We classified TS and CO conditions based on the functional connectivity networks within a given time window. Connectivity networks were constructed by averaging within- and between-region connections, followed by temporal averaging within the time windows of interest (500 ms post-S1 and S2, respectively). From the resulting symmetric 6 × 6 adjacency matrix, we extracted 21 independent features.

For classification, we performed 100-fold cross-validation, selecting a random test set of two subjects per fold. The remaining 38 subjects formed the training set, which was used for mean-centering and scaling the data to unit variance (Z-score), selecting the 10 most discriminative features based on the highest ANOVA F-values, and fitting the Linear Discriminant Analysis (LDA) classifier. Classification accuracies from the test sets were averaged across folds and reported. Due to the small dataset, we omitted a validation set and accepted data leakage while exploring hyperparameters (test set size, feature selection, and classifier choice). Consequently, reported accuracies are likely overestimated, and overfitting could not be controlled with a test set of only two samples.

As a control, we repeated the procedure with a surrogate dataset in which group labels were randomly shuffled in each fold, yielding chance-level performance (∼50 %).

### 2.12 Global efficiency and average clustering

Global efficiency was computed to assess the overall integration of the functional brain network using networkx library’s built-in function *nx.global_efficiency(G)* in Python. To quantify local segregation, the average clustering coefficient was calculated using the *nx.average_clustering(G)* function, which measures the degree to which nodes in the network tend to form tightly connected clusters. The network was constructed based on rPLVs between electrode pairs. For each subject, functional connectivity matrices were extracted for the predefined time windows [−3200, −2700] and [0, 500], following the same approach as the rPLV analysis (see Section 2.7 for details). Time indices corresponding to each window were identified, and connectivity values were averaged across time points to obtain a representative adjacency matrix.

The resulting adjacency matrix was used to construct an undirected, weighted graph *G*, where nodes represented electrodes and edge weights reflected the functional connectivity strength between them. Global efficiency was then calculated using the following formula:

$$E_{\mathrm{glob}}=\frac{1}{N(N-1)}\sum_{i\neq j}^{N}\frac{1}{L_{i,j}}$$

where *N* is the total number of nodes, and *L_i,j_* is the shortest path length between nodes *i* and *j*. Higher values of Global efficiency indicate more efficient information transfer across the network.

The clustering coefficient for a node is defined as the proportion of connections that exist between its immediate neighbors relative to the total possible number of such connections. The average clustering coefficient for the network is given by:

$$C=\frac{1}{N}\sum_{i=1}^{N}C_{i}$$

where $C_{i}$ is the clustering coefficient of node i. Higher values of the average clustering coefficient reflect a greater tendency for nodes to form tightly interconnected local clusters, indicating stronger local segregation within the network.

### 2.13 Statistical analysis

Reaction times (RT) for left- and right-hand task conditions were averaged across trials for each participant to compare behavioral performance between groups. Trials with RTs exceeding 1500 ms after stimulus onset or occurring within 150 ms post-stimulus were excluded to eliminate anticipatory and excessively delayed responses. This criterion ensured that only valid, task-related reactions were included in the final analysis. Task accuracy was calculated as the percentage of correct responses, with anticipatory responses defined as button presses occurring between the warning stimulus (S1) and the imperative stimulus (S2). For behavioral data analysis, we employed the non-parametric Mann-Whitney *U* test due to the non-normal distribution of the data, as confirmed by Shapiro-Wilk tests (*p* < 0.05).

To identify regions with significant increases in phase locking within each group (theta and alpha frequency), we performed pointwise t-tests. Differences in rPLV and global efficiency between TS and CO subjects were assessed using non-parametric Mann-Whitney U tests, given the non-normal distribution of the data. Statistical tests were performed at a significance level of *p* = 0.05 (FDR corrected). Associations between global efficiency and behavioral measures (reaction time, task accuracy, and anticipatory errors) were examined using linear regression analyses. For each group and time window, regression models were computed, and FDR correction was applied to all resulting p-values to control for multiple comparisons.

For exploratory area-based analyses, we refrained from applying FDR correction due to the prohibitive number of comparisons arising from the high dimensionality of the data. This approach would have rendered significance thresholds overly conservative, obscuring biologically plausible effects. To validate our findings, we employed complementary multivariate approaches: PCA to identify dominant network configurations, Euclidean distance metrics to quantify global group separations, classifier accuracy to assess discriminative power, and global efficiency to evaluate network integration. These methods circumvented multiple-testing limitations by focusing on system-level dynamics rather than individual connections, providing converging evidence of robust group differences in network organization.

## 3 Results

### 3.1 Behavioral task-performance

Error rates and reaction times were analyzed to investigate behavioral task performance across TS and CO subjects. Between-group comparison showed no significant differences in motor performance regarding reaction times, anticipatory responses (errors in between stimuli), or general task accuracy (see Table 1).

### 3.2 Event-related strengthening of phase coupling

Prior to the analysis of group-related connectivity differences, rPLV data were examined for statistically significant connectivity patterns within the areas of interest. This approach aims to facilitate the definition of temporal windows exhibiting robust connectivity both within and between areas of interest.

**Fig. 2:**
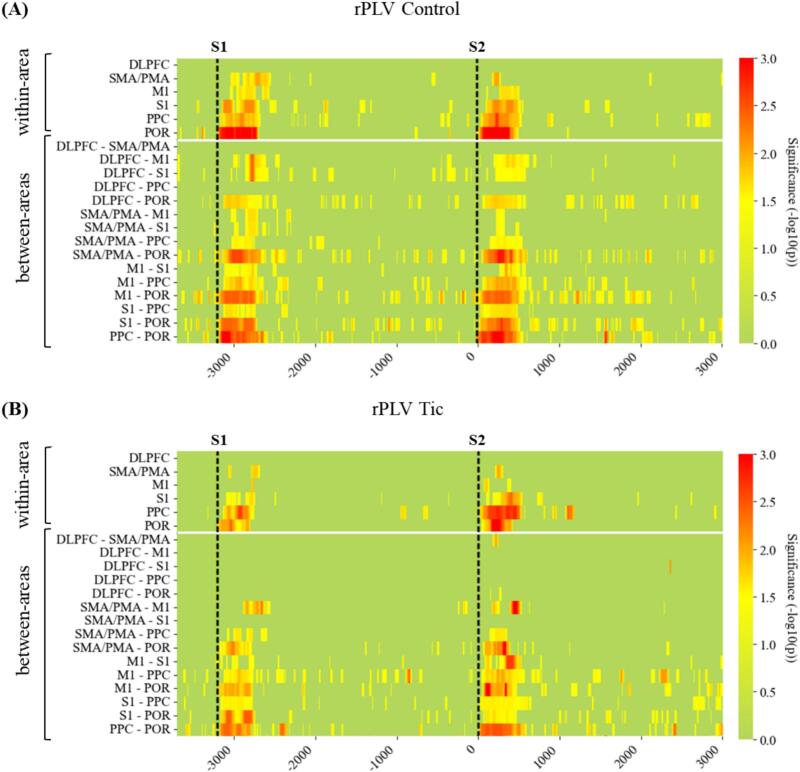
Event-related changes in theta phase locking. (A) Colormap representing statistical analysis of rPLV over time in CO and (B) TS subjects. Connectivity measures are cluster in within-area (upper part) and between-area connectivity (bottom part) of defined areas of interest. Vertical dashed lines indicate stimulus presentation of warning and imperative stimulus. Connectivity significance is represented as − log_10_(p), whereas higher values indicate more statistically reliable connectivity across subjects. *Note.* DLPFC = Dorsolateral prefrontal cortex; SMA/PMA = supplementary motor area/premotor area; M1 = primary motor cortex; S1 = primary somatosensory cortex; PPC = posterior parietal cortex; POR = parieto-occipital region.

For both groups, theta band phase locking showed a significant event-related increase, particularly in the time window of 0 to 500 ms following the warning and the imperative stimulus. Fig. 2 illustrates time points and areas with an increase in phase locking for theta band frequency that were statistically significant for either left or right motor tasks. For both groups, no significant connectivity was observed within the area of DLPFC. However, in contrast to tic subjects, control subjects showed significant phase locking between DLPFC and other regions of interest. In all other areas, significant phase synchronization was observed both within and between regions in both groups. For posterior regions (PPC and POR), the increase in rPLV was statistically most robust, indicating a stronger and more reliable connectivity pattern in these areas.

To assess the frequency specificity of these effects, we also examined connectivity in the alpha band (for illustration, see Supplementary Material, Fig. 1). In both groups, however, only minimally significant event-related phase locking was observed in the alpha frequency range during the Contingent Negative Variation. This contrasts with well-documented alpha power modulation reported in Contingent Negative Variation paradigms, highlighting a dissociation between power changes and phase-based connectivity measures (Bender et al., 2004, Güntekin et al., 2023, Schmidgen et al., 2024). Importantly, connectivity metrics such as rPLV are typically corrected for power differences, indicating that the lack of significant alpha connectivity reflects a true absence of phase synchronization rather than a power-related artifact. Notably, this pattern is consistent with developmental findings showing that, while connectivity in both theta and alpha bands increases with age, alpha connectivity in particular becomes more robust and functionally specialized later in development, and is more heterogeneous and variable during maturation, which can contribute to reduced group-level significance due to high inter-individual differences in this age range (Klimesch, 2012, Smit et al., 2012, Uhlhaas et al., 2009). However, robust theta synchronization characterizes typical task engagement in the analyzed developmental window (Cellier et al., 2021, Gander et al., 2025).

Since event-related connectivity increase in theta frequency was most pronounced within 500 ms following stimulus presentation of S1 (warning cue) and S2 (imperative cue, motor response), we focused on those specific time windows to analyze group-related differences in stimulus processing, movement preparation, and motor output. Area-based connectivity showed no pronounced differences between left and right task conditions for both groups, therefore, datasets were aggregated and used for the following analysis.

### 3.3 Differences in phase coupling between groups

**Fig. 3:**
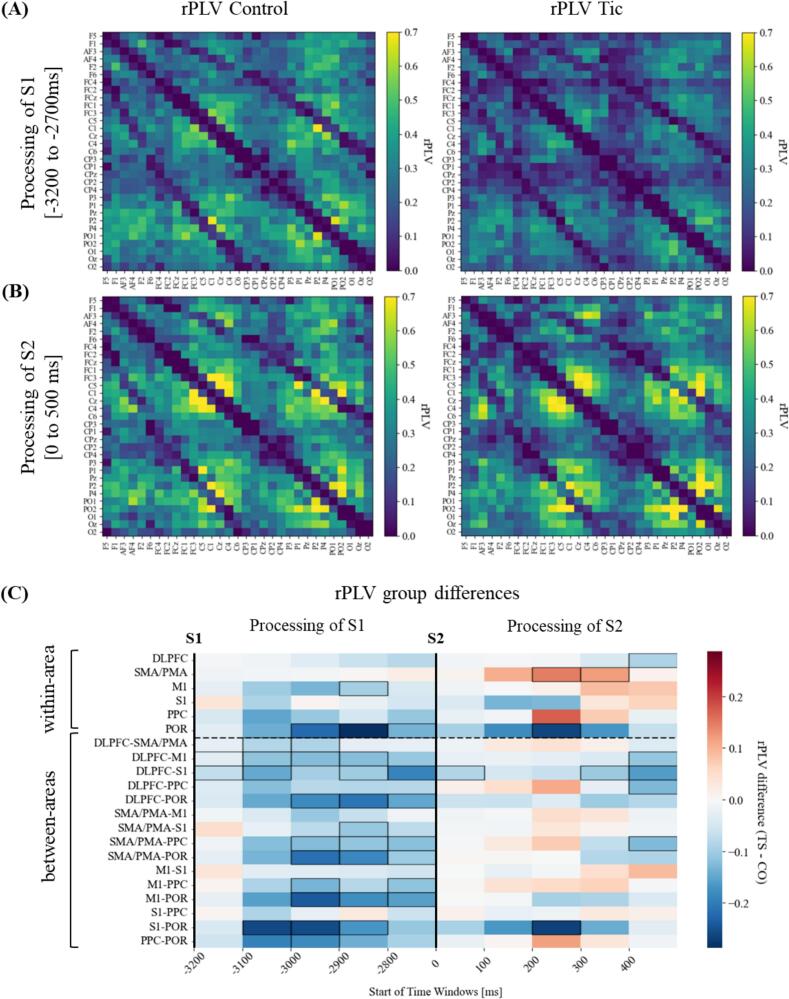
Average rPLV and differences between TS and CO across brain areas and time. (A) rPLC matrices of CO (right) and TS (left) subjects averaged across time windows of S1 processing, and (B) of S2 processing (respectively 0 to 500 ms after stimulus presentation). (C) Differences in event-related phase synchronization (rPLV) between TS and CO subjects. Data were calculated for 100 ms time windows, resulting in five time windows respectively after stimulus presentation of S1 (left) and S2 (right). rPLV connectivity was classified into connections within each brain area (upper part) and connections between the predefined brain areas (lower part). Blue squares (negative values) indicate higher connectivity in CO, and red squares show increased connectivity in TS subjects. Time intervals and areas showing significant differences between TS and CO are framed in black. Please note, rPLV differences were not FDR-corrected due to the large number of areas and tests, making correction impractical. However, consistent patterns across multiple comparisons support the robustness of the findings*. Note.* DLPFC = dorsolateral prefrontal cortex; SMA/PMA = supplementary motor area/premotor area; M1 = primary motor cortex; S1 = primary somatosensory cortex; PPC = posterior parietal cortex; POR = parieto-occipital region. (For interpretation of the references to colour in this figure legend, the reader is referred to the web version of this article.)

To provide an initial overview of rPLV differences between TS and CO, connectivity matrices were plotted for the 0–500 ms time window following S1 and S2 (Fig. 3A, B). These matrices represent group-averaged functional connectivity across all subjects within each group for all electrodes within regions of interest. Visual inspection reveals distinct differences in overall connectivity patterns between TS and CO, with notable variations in the strength and distribution of functional connections, especially in the time window following S1. Average phase synchronization across all electrodes showed significantly reduced connectivity in TS subjects for S1 processing (Mdn = 0.22, IQR = 0.06; CO: Mdn = 0.30, IQR = 0.14; U = 98.000, Z = −2.76, *p* = 0.012, r = 0.44). Overall connectivity strengths within the time window of S2 processing did not show significant group differences (TS: Mdn = 0.31, IQR = 0.15; CO: Mdn = 0.36, IQR = 0.10; U = 165.000, Z = −0.95, *p* = 0.351, r = 0.15).

To validate differences in connectivity patterns, area-based analyses were conducted. Tic-related deviations in phase synchronization were examined by subdividing regions of interest into within- and between-area connectivity. To capture dynamic changes, data were segmented into five 100-ms subintervals for S1 and S2 (Fig. 3C). Mann-Whitney U tests revealed significant between-group differences in event-related connectivity (for detailed statistics, see Supplementary Material, Table 1). TS participants showed reduced phase synchronization within and between most brain areas after S1, with the strongest and most robust reductions in the POR area. While rPLV strengths in other regions also indicated tic-related reductions, within-area connectivity loss was less statistically consistent than the more pronounced between-area reductions across all regions of interest.

Processing of S2 elicited less pronounced differences in synchronization between groups. Reductions in rPLV strength were mainly observed between the DLPFC and S1, as well as between S1 and POR. Notably, TS participants showed a more pronounced increase in phase synchronization within the SMA/PMA (for detailed statistics see Supplementary Material, Table 2).

### 3.4 Assessing network differences with PCA, euclidean distance and classification

Leveraging PCA and Euclidean distance measures, we quantified global network disparities between groups, providing a dimensionality-reduced representation of connectivity changes associated with stimulus processing.

**Fig. 4:**
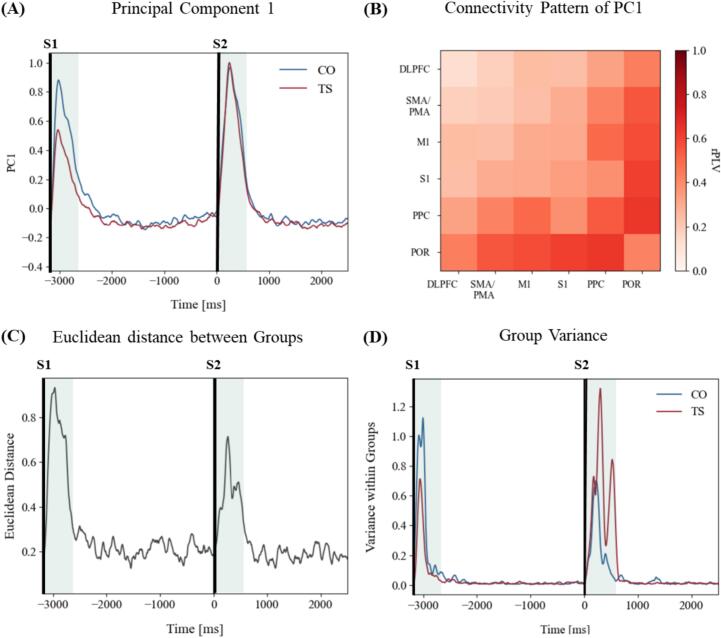
Network differences. (A) Time course of PC1 for TS (red) and CO (blue), with a pronounced increase within 500 ms time windows after stimulus presentation. TS shows reduced PC1 values for the processing period of S1. (B) The connectivity pattern of PC1 shows an overall increase in phase synchronization, most pronounced for parietal areas. (C) Euclidean distance showed noticeable group separations after both S1 and S2, with the largest difference occurring after S1. (D) Variance within the group for TS (red) and CO (blue), with higher variance for CO after S1 and higher variance for TS after S2. (For interpretation of the references to colour in this figure legend, the reader is referred to the web version of this article.)

In the PCA analysis, Principal Component 1 (PC1) accounted for 30 % of the variance in the dataset (Fig. 4A, B). The eigenvector matrix revealed that PC1 was associated with an increase in connectivity, as reflected by higher rPLVs in the original data. Notably, all other components accounted individually for less than 3 % of the variance, highlighting that PC1 played a dominant role in explaining the patterns of connectivity in the datasets. Analysis of PC1 scores revealed distinct temporal patterns between groups (Fig. 4A). Following the warning stimulus (S1), CO subjects exhibited higher PC1 values compared to TS subjects, indicating a more pronounced increase in overall connectivity during S1 processing. However, after the imperative stimulus (S2), PC1 values converged between the groups, suggesting a reduction in the disparity of connectivity strengths.

Complementing the PCA findings, Euclidean distance analysis quantified the overall separation of neural states between groups. As expected, the most pronounced group differences were observed within 500 ms following both stimuli, with the largest separation occurring after S1 (for details see Fig. 4C). This confirms that the critical divergences in network configuration between TS and CO groups are most evident in the immediate post-stimulus periods. While group separation strongly decreased during the inter-stimulus interval, a clear distinction remained after S2, albeit less pronounced than post-S1. Variance analysis revealed greater processing variance in the CO group after S1, while the TS group exhibited higher processing variance after S2 (Fig. 4D).

To assess the discriminative power of these network differences, we employed a classifier analysis. The classifier achieved an accuracy of 76.5 % in the time window following S1, and 72.5 % after S2. These results exceeded chance levels, as demonstrated by shuffled accuracy rates of 52.0 % for S1 and 49.0 % for S2. This classification performance further validates the presence of distinct network patterns between TS and CO groups during stimulus processing phases.

### 3.5 Global efficiency and local clustering between groups

**Fig. 5:**
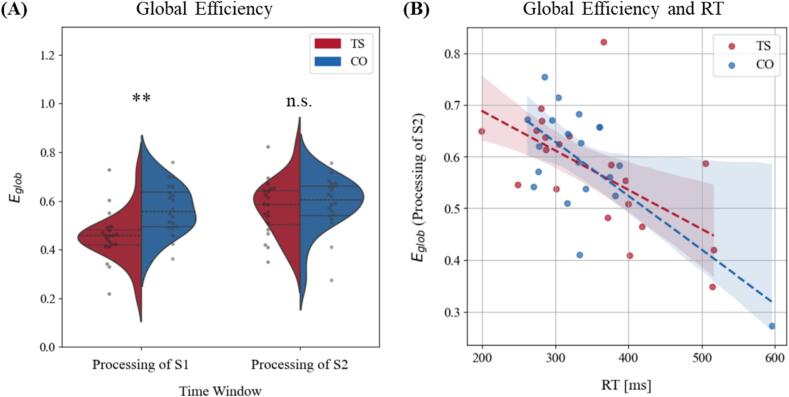
Global efficiency (A) Global Efficiency (E_glob_) comparison between TS and CO for Processing of S1 and S2. Data are visualized using violin plots, with overlaid swarm plots to show individual subject data points. The statistical threshold (** *p* ≤ 0.01) has been corrected for multiple comparisons using FDR*.* (B) Relationship between motor performance and Global efficiency (E_glob_) post S2. RT was significantly correlated with the Global efficiency of S2 processing (TS: *p* = 0.008; CO: *p* = 0.004; FDR corrected).

To determine whether connectivity differences − particularly the reduced connectivity observed in TS during S1 processing − affected network efficiency, we analyzed global efficiency following both S1 and S2 (Fig. 5A). Group comparison reveald significantly reduced efficiency during S1 processing in TS subjects (TS: Mdn = 0.46, IQR = 0.06; CO: Mdn = 0.56, IQR = 0.15; U = 77.000, Z = −3.33, *p* = 0.002, r = −0.53). During processing of S2, groups showed comparable network efficiency (TS: Mdn = 0.59, IQR = 0.14; CO: Mdn = 0.6, IQR = 0.12; U = 168.000, Z = −0.87, *p* = 0.394, r = −0.14).

In addition to global efficiency, we analyzed local segregation to assess the extent to which functional brain networks are organized into tightly interconnected local clusters, which is thought to support specialized information processing and efficient local communication. Comparable to global efficiency, group comparison revealed significantly reduced local segregation, as measured by the average clustering coefficient, during S1 processing in TS subjects (TS: Mdn = 0.17, IQR = 0.16; CO: Mdn = 0.33, IQR = 0.20; U = 76.000, Z = −3.35, *p* = 0.002, r = −0.53). During S2 processing, groups showed comparable clustering coefficients (TS: Mdn = 0.31, IQR = 0.20; CO: Mdn = 0.37, IQR = 0.14; U = 176.000, Z = −0.65, *p* = 0.525, r = −0.10, for illustration, see Supplementary Material, Fig. 2).

### 3.6 Global efficiency across motor performance

Network efficiency following S2 correlated significantly with reaction time in both groups (TS: R^2^ = 0.374, F(1, 18) = 10.77, *p* = 0.008; CO: R^2^ = 0.467, F(1, 18) = 15.74, *p* = 0.003), indicating that higher efficiency was linked to faster responses (Fig. 5B). In contrast, there was no evidence of a relationship between network efficiency during S1 processing and reaction time in either group (TS: R^2^ = 0.073, F(1, 18) = 1.41, *p* = 0.251; CO: R^2^ = 0.177, F(1, 18) = 3.87, *p* = 0.086). Task accuracy and anticipatory errors also showed no evidence of a relationship with network efficiency in either group or time window (for detailed statistics, see Supplementary Material, Table 3).

**Table 3:** Task-performance parameters in TS and CO group.

Performance	TS subjects	CO subjects	Statistics	*P*
RT (left) [Mdn; IQR]	346.70; 97.57	326.10; 69.94	U = 224.00, Z = 0.65, r = 0.10	0.53
RT (right) [Mdn; IQR]	329.40; 129.11	334.75; 67.44	U = 195.00, Z = −0.14, r = 0.02	0.90
Anticipatory response [Mdn; IQR]	8.00; 9.50	7.00; 5.50	U = 219.50, Z = 0.53, r = 0.08	0.61
Accuracy [Mdn; IQR]	89.50; 9.75	92.00; 4.5	U = 166.50, Z = −0.91, r = 0.14	0.37

*Note.* RT = Reaction-time, Mdn = Median, IQR = Interquartile range.

## 4 Discussion

This study examined functional connectivity during sensorimotor integration, motor preparation, and execution in children and adolescents with TS. Our findings revealed widespread alterations in theta-band connectivity across frontal, central, parietal, and occipital brain regions. The most pronounced differences emerged during the processing of the warning stimulus (S1), marked by a tic-related reduction in theta-band network synchronization. Distinct group differences were also observed during the processing of the imperative stimulus (S2), characterized by reorganized network patterns rather than diminished connectivity.

Both groups displayed the strongest phase coupling immediately after stimulus presentation, lasting for approximately 500 ms. The stimuli triggered a synchronized neural response across multiple brain regions, consistent with previous studies on visual-attentional processing in children (Santhana Gopalan et al., 2019, Zarka et al., 2021). During the first few hundred milliseconds, several key cognitive processes are engaged, including sensory processing, attentional allocation, information integration, motor preparation, resource distribution, and top-down modulation (Madl et al., 2011, Tan et al., 2024). These rapid and interconnected processes underscore the complex neural dynamics involved in stimulus processing and response preparation.

Neuronal networks arose across all examined brain regions. However, while healthy subjects exhibited modest connectivity between the DLPFC and other brain regions, subjects with TS showed nearly no significant integration of the DLPFC, highlighting distinct differences in network architecture between the groups. Previous studies showed an age-related increase of DLPFC activation (Achterberg et al., 2020), alongside a developmental increase of functional coupling to other brain regions involved in motor control (Steinbeis et al., 2014, Uddin et al., 2011). In line with our data, studies focusing on tic pathophysiology in children reported a reduced involvement of the frontal-parietal networks (Church et al., 2009, Fan et al., 2018). Fair et al. (2009) observed widespread differences in functional connectivity between frontoparietal control networks and suggested that reduced efficiency in TS is particularly pronounced between distant brain regions, suggesting a tic-related functional immaturity.

### 4.1 Phase coupling following S1

Group differences in theta connectivity were most pronounced during the processing of the informative warning cue of our pre-cued reaction time task. The data suggest an extensive deviation in theta connectivity across all investigated brain regions. Besides a pronounced reduction in overall phase synchronization, subjects with TS showed a reduced network efficiency and local clustering. This finding aligns with the growing understanding of TS as a network disorder rather than a condition affecting isolated brain regions.

Reduced local clustering in brain networks indicates a lower tendency for brain regions to form tightly interconnected local groups, which are thought to support specialized information processing and efficient local communication (Liao et al., 2017, Masuda et al., 2018). A high clustering coefficient is an indicator of a small-world network organization, facilitating both local specialization and global integration. In typical development, the maturation of brain networks is characterized by increasing small-world properties, reflecting more efficient and flexible information processing as the brain develops (Bullmore and Sporns, 2009, Fair et al., 2009, Liao et al., 2017). Reduced local clustering during sensorimotor integration and stimulus processing in TS may reflect an immature network organization, specifically during these processes, rather than a general network deficit, as clustering coefficients showed normal values during movement preparation and execution. This pattern suggests a transient disruption in local network specialization when processing task-relevant sensory information.

Overall, the reduced network measures might reflect difficulties in efficiently allocating attentional resources during the preparatory phase. Furthermore, our analysis revealed reduced variability in network configurations (as measured by Euclidean distance) in TS subjects following S1. This decreased variability suggests constrained neural flexibility during stimulus processing. Notably, TS subjects achieved normal response execution efficiency during S2 processing, despite initial preparatory differences. They showed no impairment in motor performance regarding reaction times, accuracy, and especially anticipatory responses (premature button press after presentation of warning stimulus). These findings are consistent with other studies reporting no tic-related deficits in task performance (Marsh et al., 2007, Schmidgen et al., 2023, Wylie et al., 2016). However, in contrast to our results, other research has demonstrated that individuals with TS exhibit impairments and reduced performance in visual processing, particularly in tasks requiring visuomotor integration (Brookshire et al., 1994, Schultz et al., 1998). The recruitment of parietal and frontal brain areas is essential for the dynamic process of visual feature integration (Castellano et al., 2014), involving top-down modulation from these regions. It cannot be ruled out that the motor task paradigm may not be sensitive enough to detect subtle differences in performance that might be present in more complex or demanding tasks.

Adelhöfer et al. (2021) found increased neural noise levels (1/*f* noise) during sensorimotor processing in children and adults with Tourette Syndrome. Increased neural noise is associated with reduced synchronization of neuronal activity (Voytek et al., 2015). Higher noise levels during stimulus processing likely impair area synchronization and neuronal communication (González-Villar et al., 2017), suggesting TS-related alterations in neural dynamics. Sun et al. (2020) reported a stronger reduction of somatosensory evoked potentials 15 min after a rTMS protocol application (1 Hz, 90 % of the resting motor threshold) in TS patients. They suggested the motor-sensory cortex circuit to be responsible for the suppression of the sensory system. This interpretation supports the view of TS as a sensorimotor disorder rather than solely a motor dysfunction. In an fMRI study by Atkinson-Clement et al. (2020), adult TS patients with intermittent explosive outbursts showed reduced connectivity within the sensory-motor cortico-basal ganglia network that was related to difficulties in sensorimotor integration, action selection, and decision-making. The findings align with our results, indicating reduced connectivity and network efficiency, particularly following the presentation of the informative S1 cue, which demanded greater sensory processing and motor preparation compared to S2 processing.

### 4.2 Perception-action binding

However, it is also plausible that these deviations represent a compensatory mechanism rather than a deficit, especially given that tic patients did not exhibit impaired motor performance. The reduced connectivity could reflect a compensatory reorganization aimed at weakening perception–action binding to prevent premature motor output. Research further suggests a compensatory downregulation in response to urges and heightened sensitivity to external stimuli (Friedrich et al., 2021).

Children with TS showed enhanced perceptual-motor sequence learning, likely due to strengthened connections between stimuli and motor responses (Shephard et al., 2019, Takács et al., 2018). This supports the idea that tic-related mechanisms may enhance habit formation (Delorme et al., 2016, Singer, 2016), indicating an increased tendency to form links between sensory cues and motor actions. The cognitive framework of the theory of event coding (TEC) explains how perception (stimulus) and action (response) are integrated within the brain (Hommel et al., 2001). The framework is based on the assumption that perception and action share a common representational format and that the binding information is stored in a binding “event file”. This mechanism allows stimuli with shared features to trigger similar actions more efficiently, facilitating habit learning. Such associations influence subsequent stimulus processing and may contribute to automatic action triggering in TS (Kleimaker et al., 2020, Petruo et al., 2019). By weakening the perception–action binding, this neural adaptation may help TS patients prevent premature motor output and exert better control over their tics. This compensatory reorganization aligns with the observed enhanced perceptual-motor learning and increased habit formation in TS, suggesting a complex interplay between altered neural connectivity and behavioral adaptations in managing tic symptoms.

### 4.3 Phase coupling following S2

In contrast to S1 processing, the neural networks engaged during S2 processing showed more subtle group differences, characterized by shifts in network organization rather than changes in overall connectivity strength. Notably, children with TS demonstrated increased phase coupling both within and between several brain regions. Although the increase in synchronization of individual areas was largely not statistically significant, analyses of PCA and Euclidean distance revealed that global network dynamics differed significantly between groups. These multivariate approaches captured subtle yet consistent alterations in overall connectivity patterns, highlighting the importance of considering network-level changes in Tourette Syndrome that may not be apparent when examining individual connections in isolation. Interestingly, Euclidean distance analysis revealed a striking contrast in network variability for TS subjects across task phases. Following S1, variability was reduced, whereas after S2, TS subjects showed increased variability compared to controls. This heightened variability after the imperative stimulus suggests a potential “release” from the constrained neural states seen during the preparatory phase. Such a shift may indicate a transition from rigid control during preparation to more flexible, yet potentially less stable, network dynamics when responding rapidly. However, this compensatory mechanism may come at the cost of reduced neural flexibility, potentially limiting the ability to adapt to more complex or demanding tasks. Importantly, the processing networks of both groups achieved comparable efficiency. These results align with the idea that TS involves more complex alterations in preparatory and control processes rather than in basic motor execution mechanisms (Beste and Münchau, 2018, Mielke et al., 2021).

While the overall differences in imperative stimulus processing were less pronounced, a closer examination of specific brain regions revealed interesting patterns. Phase synchronization data suggest a significantly increased functional coupling within the SMA and premotor areas during late motor preparation and execution (200 to 400 ms post S2). Many studies suggested deviations within the SMA to be a crucial factor within tic-related pathophysiological mechanisms. Especially, it was shown that the SMA is actively involved in tic generation (Bloch et al., 2006, Hampson et al., 2009, Neuner et al., 2014). Applying 1  Hz inhibitory rTMS to the SMA resulted in reduced tic frequency, implying that excessive SMA activity is directly linked to tic manifestation (Le et al., 2013, Mantovani et al., 2007, Wu et al., 2014). Besides a tic-related overactivation of the SMA, research has revealed enhanced connectivity within other areas related to SMA and premotor areas (Biswal et al., 1998, Fattapposta et al., 2005). These findings are consistent with our phase coupling data, underscoring the critical role of SMA hyperconnectivity in the pathophysiology of Tourette syndrome and its potential as a target for therapeutic interventions.

### 4.4 Global efficiency and task performance

The efficiency of neural networks involved in processing S1 did not correlate with reaction times, suggesting that network efficiency during the preparatory phase may not directly influence motor response speed. In contrast, network efficiency following S2 showed a significant correlation with reaction times in both groups, indicating that greater global efficiency during imperative stimulus processing and response execution is directly linked to faster motor responses. These findings suggest that early preparatory processes may be less critical for determining motor performance than the efficiency of later stages of stimulus processing and action execution. Overall, the results underscore the importance of temporal dynamics in neural processing for predicting behavioral performance.

Global efficiency of brain networks, particularly within the motor system, has been linked to both motor performance and symptom severity in movement disorders, with higher efficiency generally associated with better performance and reduced motor symptoms (Li et al., 2022, Novaes et al., 2021, Xiao et al., 2016). The observed correlation between network efficiency and reaction time in both groups suggests that this relationship represents a fundamental aspect of motor control. This aligns with the notion that efficient information processing across brain networks is essential for rapid and accurate motor responses. Moreover, the preserved association between network efficiency and motor performance in TS subjects suggests that core motor control processes remain intact despite the presence of tics.

However, while our results indicate normal task performance during a structured laboratory environment, it is important to note that this may not accurately represent the everyday experiences of individuals with TS. These laboratory settings typically involve focused, goal-directed activities that are known to reduce tic frequency and improve the regulation of movements, likely by engaging compensatory mechanisms (Jackson et al., 2011, Metzlaff et al., 2022, Schüller et al., 2018). Neural adaptations may allow individuals with TS to achieve normal performance in structured settings, but may also come at the cost of increased cognitive effort. In contrast, during daily life, when sustained attention is less easily maintained and concentration fluctuates, the control over motor outputs and the ability to suppress tics remain limited. Nevertheless, compensatory processes may explain the significant reduction in tic frequency observed during adolescence, as the maturation of cognitive control networks could enhance the effectiveness of these adaptive strategies over time (Ricketts et al., 2022).

Our results suggest that interventions should not only target tic suppression but also support the broader neural networks involved in cognitive and motor control. Specifically, interventions could be developed to strengthen the adaptive modulation of perception–action binding, for example, through cognitive-behavioral strategies or neurofeedback protocols that train children to flexibly adjust their sensorimotor integration in response to external stimuli (Petruo et al., 2020). By providing such targeted training, it may be possible to reduce the cognitive effort required for controlling motor outputs and enable children to apply these strategies more effectively in everyday life.

## 5 Limitations

While our study provides valuable insights into neural network efficiency and motor performance in Tourette Syndrome, several limitations should be considered when interpreting the results. (1) Our study included a relatively small sample size, which may have reduced statistical power, potentially obscuring subtle effects or relationships. (2) While EEG provides excellent temporal resolution, its spatial resolution is limited. Complementary neuroimaging techniques, such as fMRI, could provide additional spatial information about network dynamics. (3) Our cross-sectional design provides a developmental snapshot, but longitudinal studies are essential to capture the dynamic changes in TS over time, particularly concerning symptom persistence or remission in adulthood. This would allow for a deeper understanding of developmental trajectories and compensatory mechanisms in TS.

## 6 Conclusion

TS patients exhibit reduced theta connectivity, network efficiency, and local clustering during the processing of the informative warning cue (S1), indicating disruptions in sensorimotor integration. The widespread deviations across all investigated brain regions suggest that network alterations in TS extend beyond the traditional cortico-striato-thalamo-cortical circuits, reflecting a more extensive reorganization of brain dynamics. Notably, the reduced connectivity during S1 processing may represent a compensatory mechanism aimed at weakening perception–action binding, potentially preventing premature motor output and aiding in tic control. During imperative stimulus (S2) processing, TS patients showed increased functional coupling within the SMA and premotor areas during late motor preparation and execution, aligning with prior research identifying these regions as central to tic generation and control. Importantly, TS patients demonstrated comparable network efficiency during S2 processing and preserved motor performance, supporting the view that basic motor execution mechanisms remain largely intact in TS. These findings underscore the complex neural network dysfunctions underlying TS, particularly in task-related processing and sensorimotor integration. Altered theta connectivity patterns may reflect compensatory adaptations or fundamental differences in how individuals with TS process and integrate sensory information.

## CRediT authorship contribution statement

**Julia Schmidgen:** Investigation, Data curation, Visualization, Writing – original draft, Validation, Conceptualization, Methodology, Formal analysis. **Theresa Valentine Heinen:** Methodology, Conceptualization, Writing – review & editing, Investigation. **Felix Schmitt:** Validation, Formal analysis. **Kerstin Konrad:** Resources, Project administration, Conceptualization, Writing – review & editing, Funding acquisition. **Stephan Bender:** Validation, Funding acquisition, Supervision, Methodology, Conceptualization, Writing – review & editing, Project administration, Resources.

## Funding

This work was funded by the 10.13039/501100001659Deutsche Forschungsgemeinschaft (DFG, German Research Foundation) – SFB 1451 – Project-ID 431549029.

## Declaration of competing interest

The authors declare that they have no known competing financial interests or personal relationships that could have appeared to influence the work reported in this paper.

## Data Availability

The code used for data analysis is available at https://github.com/JuliSchmidgen/rPLV-Connectivity-Analysis, to allow readers to review and revise the code. Due to ethical considerations, the data supporting the findings of this study are available upon reasonable request from the corresponding author. The data cannot be used with the code for reproduction of results until they are made available, which will be subject to ethical guidelines. Any requests for data access will be processed in accordance with the relevant procedures, including formal data-sharing agreements or approval from the appropriate ethics committee.
