# Supplementary material for: Altered network connectivity and global efficiency in tourette syndrome: insights into sensorimotor integration
Source: Neuroimage Clin. 2025 Jul 13;48:103845. doi: 10.1016/j.nicl.2025.103845 (PMC12296517; doi:10.1016/j.nicl.2025.103845)
Supplement: Supplementary Data 1 [file mmc1.docx]

# Supplementary materials


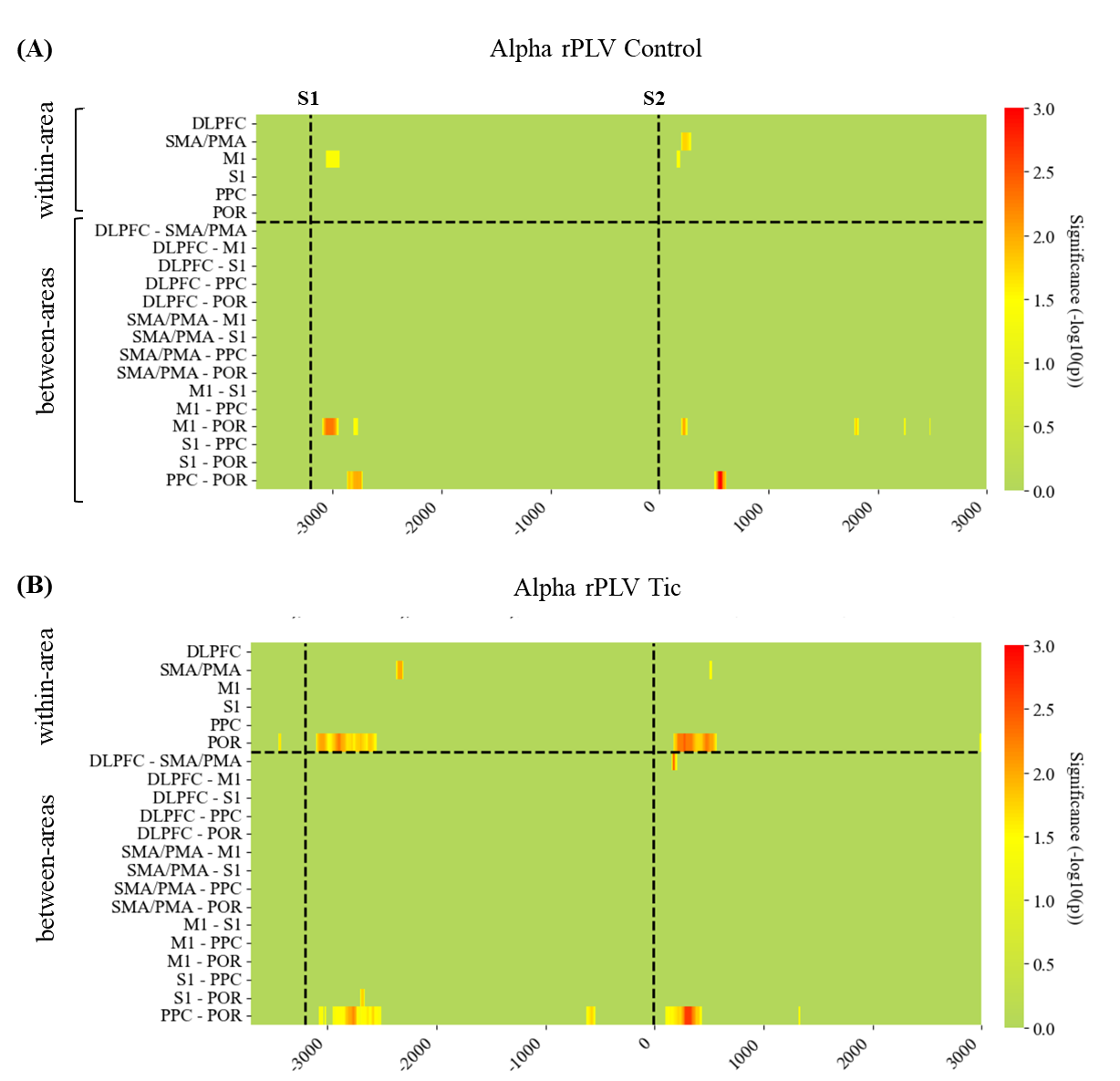


Fig 1 Event-related changes in alpha phase locking. (A) Colormap representing statistical analysis of rPLV in alpha frequency over time in CO and (B) TS subjects. Connectivity measures are cluster in within-area (upper part) and between-area connectivity (bottom part) of defined ROIs. Vertical dashed lines indicate stimulus presentation of warning and imperative stimulus. Connectivity significance is represented as −log_10_​(p), whereas higher values indicate more statistically reliable connectivity across subjects. *Note.* DLPFC = Dorsolateral prefrontal cortex; SMA/PMA = supplementary motor area/premotor area; M1 = primary motor cortex; S1 = primary somatosensory cortex; PPC = posterior parietal cortex; POR = parieto-occipital region.

Table 1 Significant connectivity differences between groups during S1 processing. Please note that rPLV differences were not FDR-corrected due to the large number of regions and tests, making correction impractical.

| Typ | Area | Time window | Mdn [IQR] TS | Mdn [IQR] CO | U | *p* - value | r |
| --- | --- | --- | --- | --- | --- | --- | --- |
| Within Area | M1 | -2900 to -2800 | 0.128 [0.189] | 0.238 [0.159] | 124 | .041 | .31 |
|  | POR | -3000 to -2900 | 0.144 [0.318] | 0.438 [0.372] | 112 | .018 | .28 |
|  | POR | -2900 to -2800 | 0.182 [0.299] | 0.510 [0.302] | 81 | .001 | .2 |
|  | POR | -2800 to -2700 | 0.030 [0.129] | 0.159 [0.154] | 92 | .004 | .23 |
| Between Area | DLPFC – SMA/PMA | -3100 to -3000 | 0.081 [0.123] | 0.162 [0.169] | 117 | .026 | .29 |
|  | DLPFC – SMA/PMA | -3000 to -2900 | 0.126 [0.147] | 0.251 [0.101] | 124 | .041 | .31 |
|  | DLPFC – M1 | -3200 to -3100 | 0.046 [0.032] | 0.070 [0.073] | 127 | .049 | .32 |
|  | DLPFC – M1 | -3100 to -3000 | 0.102 [0.147] | 0.218 [0.160] | 101 | .008 | .25 |
|  | DLPFC – M1 | -3000 to -2900 | 0.133 [0.180] | 0.242 [0.214] | 110 | .016 | .28 |
|  | DLPFC – M1 | -2900 to -2800 | 0.153 [0.190] | 0.257 [0.207] | 109 | .015 | .27 |
|  | DLPFC – S1 | -3200 to -3100 | 0.002 [0.110] | 0.072 [0.128] | 115 | .022 | .29 |
|  | DLPFC – S1 | -3100 to -3000 | 0.040 [0.179] | 0.167 [0.149] | 100 | .007 | .25 |
|  | DLPFC – S1 | -2900 to -2800 | 0.139 [0.201] | 0.234 [0.207] | 126 | .047 | .32 |
|  | DLPFC – S1 | -2800 to -2700 | 0.151 [0.155] | 0.346 [0.194] | 78 | .001 | .2 |
|  | DLPFC – POR | -3000 to -2900 | 0.349 [0.285] | 0.467 [0.220] | 123 | .039 | .31 |
|  | DLPFC - POR | -2900 to -2800 | 0.237 [0.230] | 0.392 [0.303] | 99 | .007 | .25 |
|  | DLPFC - POR | -2800 to -2700 | 0.154 [0.117] | 0.289 [0.305] | 117 | .026 | .29 |
|  | SMA/PMA – S1 | -2900 to -2800 | 0.137 [0.230] | 0.302 [0.198] | 101 | .008 | .25 |
|  | SMA/PMA - PPC | -3000 to -2900 | 0.300 [0.203] | 0.388 [0.178] | 108 | .013 | .27 |
|  | SMA/PMA - PPC | -2900 to -2800 | 0.183 [0.219] | 0.301 [0.181] | 114 | .02 | .29 |
|  | SMA/PMA - PPC | -2800 to -2700 | 0.305 [0.212] | 0.369 [0.180] | 109 | .014 | .28 |
|  | SMA/PMA - POR | -3000 to -2900 | 0.335 [0.333] | 0.637 [0.438] | 117 | .027 | .29 |
|  | SMA/PMA - POR | -2900 to -2800 | 0.301 [0.282] | 0.510 [0.241] | 101 | .008 | .25 |
|  | SMA/PMA - POR | -2800 to -2700 | 0.220 [0.153] | 0.319 [0.180] | 126 | .047 | .32 |
|  | M1 – PPC | -3000 to -2900 | 0.333 [0.237] | 0.481 [0.260] | 121 | .034 | .3 |
|  | M1 – PPC | -2800 to -2700 | 0.181 [0.127] | 0.296 [0.166] | 115 | .022 | .29 |
|  | M1 – POR | -3000 to -2900 | 0.347 [0.174] | 0.632 [0.300] | 86 | .002 | .22 |
|  | M1 – POR | -2900 to -2800 | 0.376 [0.220] | 0.492 [0.292] | 114 | .021 | .29 |
|  | M1 – POR | -2800 to -2700 | 0.209 [0.137] | 0.316 [0.182] | 92 | .003 | .23 |
|  | S1 – POR | -3100 to -3000 | 0.462 [0.279] | 0.768 [0.587] | 125 | .044 | .31 |
|  | S1 – POR | -3000 to -2900 | 0.274 [0.300] | 0.571 [0.390] | 118 | .028 | .3 |
|  | S1 – POR | -2800 to -2700 | 0.274 [0.347] | 0.437 [0.343] | 121 | .034 | .31 |
|  | PPC –POR | -3000 to -2900 | 0.294 [0.317] | 0.660 [0.406] | 118 | .028 | .29 |

*Note.* DLPFC = **d**orsolateral prefrontal cortex; SMA/PMA = supplementary motor area/premotor area; M1 = primary motor cortex; S1 = primary somatosensory cortex; PPC = posterior parietal cortex; POR = parieto-occipital region.


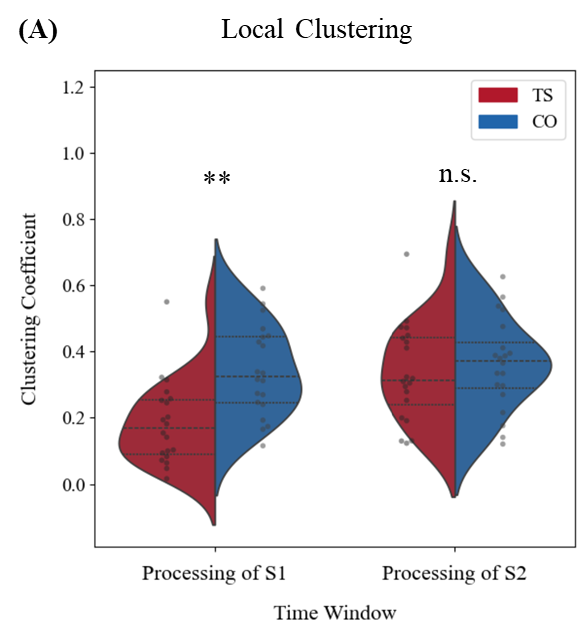


Fig 2 Local Clustering (A) Average local clustering comparison between TS and CO for processing of S1 and S2. Data are visualized using violin plots, with overlaid swarm plots to show individual subject data points. The statistical threshold (** *p* ≤ .01) has been corrected for multiple comparisons using FDR*.*

Table 2 Significant connectivity differences between groups during S2 processing. Please note that rPLV differences were not FDR-corrected due to the large number of regions and tests, making correction impractical.

| Typ | Area | Time window | Mdn [IQR] TS | Mdn [IQR] CO | U | *p* - value | r |
| --- | --- | --- | --- | --- | --- | --- | --- |
| Within | DLPFC | 400 to 500 | 0.078 [0.171] | 0.183 [0.164] | 117 | .026 | .29 |
|  | SMA/PMA | 200 to 300 | 0.165 [0.220] | 0.099 [0.173] | 123 | .041 | .31 |
|  | SMA/PMA | 300 to 400 | 0.275 [0.186] | 0.186 [0.224] | 125 | .044 | .31 |
|  | POR | 200 to 300 | 0.374 [0.316] | 0.618 [0.583] | 125 | .044 | .31 |
| Between | DLPFC – M1 | 400 to 500 | 0.253 [0.165] | 0.341 [0.144] | 118 | .028 | .3 |
|  | DLPFC – S1 | 0 to 100 | 0.080 [0.095] | 0.139 [0.165] | 125 | .044 | .31 |
|  | DLPFC – S1 | 300 to 400 | 0.166 [0.194] | 0.331 [0.201] | 111 | .017 | .28 |
|  | DLPFC – S1 | 400 to 500 | 0.161 [0.226] | 0.398 [0.293] | 97 | .006 | .24 |
|  | DLPFC - PPC | 400 to 500 | 0.184 [0.164] | 0.378 [0.219] | 108 | .013 | .27 |
|  | SMA/PMA - PPC | 400 to 500 | 0.203 [0.203] | 0.351 [0.194] | 119 | .029 | .3 |
|  | S1 – POR | 200 to 300 | 0.483 [0.390] | 0.706 [0.523] | 115 | .022 | .29 |

*Note.* DLPFC = **d**orsolateral prefrontal cortex; SMA/PMA = supplementary motor area/premotor area; M1 = primary motor cortex; S1 = primary somatosensory cortex; PPC = posterior parietal cortex; POR = parieto-occipital region.

Table 3 Regression results for associations between network efficiency and behavioral measures (task accuracy and anticipatory errors) in the TS and control groups, for both S1 (PRE) and S2 (POST) time windows.

| Outcome Variable | Time-Window | Group | R^2^ | F | *p* - value |
| --- | --- | --- | --- | --- | --- |
| Task accuracy | PRE | TS | 0.000 | 0.01 | .939 |
|  |  | CO | 0.001 | 0.02 | .939 |
|  | POST | TS | 0.01 | 0.18 | .939 |
|  |  | CO | 0.028 | 0.51 | .939 |
| Anticipatory Errors | PRE | TS | 0.000 | 0.000 | .945 |
|  |  | CO | 0.002 | 0.04 | .945 |
|  | POST | TS | 0.009 | 0.16 | .945 |
|  |  | CO | 0.045 | 0.85 | .945 |

*Note.* TS = Tourette syndrome; CO = control group; PRE = S1 time window; POST = S2 time window; FDR corrected p-values.
